# Beyond Choice: Affective Representations of Economic and Moral Decisions

**DOI:** 10.3390/bs15040558

**Published:** 2025-04-21

**Authors:** Jongwan Kim, Chaery Park

**Affiliations:** Department of Psychology, Jeonbuk National University, Jeonju-si 54896, Republic of Korea; sweet0527@jbnu.ac.kr

**Keywords:** ultimatum game, trolley dilemma, multidimensional scaling, classification, fairness, affect

## Abstract

Decision-making in economic and moral contexts involves complex affective processes that shape judgments of fairness, responsibility, and conflict resolution. While previous studies have primarily examined behavioral choices in economic games and moral dilemmas, less is known about the underlying affective structure of these decisions. This study investigated how individuals emotionally represent economic (ultimatum game) and moral (trolley dilemma) decision-making scenarios using multidimensional scaling (MDS) and classification. Participants rated their emotional responses, including positive (pleased, calm, happy, peaceful) and negative (irritated, angry, gloomy, sad, fearful, anxious) affective states, to 16 scenarios varying by game type, the presence or absence of conflict, and intensity. MDS revealed two primary affective dimensions of distinguishing conflict from no-conflict and economic from moral scenarios. No-conflict–economic scenarios were strongly associated with positive affective responses, while the no-conflict–moral scenarios elicited heightened fear and anxiety rather than positive emotions. Increasing unfairness in the ultimatum game affected affective representation, while variations in the number of lives at stake in the trolley dilemma did not. Cross-participant classification analyses demonstrated that game type and conflict conditions could be reliably predicted from affective ratings, indicating systematic and shared emotional representations across participants. These findings suggest that economic and moral decisions evoke distinct affective structures, with fairness modulating conflict perception in economic contexts, while moral decisions remain affectively stable despite changes in intensity.

## 1. Introduction

People navigate complex decisions that require balancing fairness, self-interest, and moral responsibility, whether negotiating salaries, splitting a restaurant bill, or making ethical choices that impact others. These economic and moral decisions are fundamental to social interactions, shaping relationships, trust, and societal norms (Suneja & Das, 2024; Evans et al., 2021). Prior research has demonstrated that economic decision-making is closely linked to cooperative behavior and trust, with individuals often relying on implicit social norms when engaging in bargaining or negotiation (Balliet & Van Lange, 2013). Understanding how individuals process and respond to such dilemmas is crucial not only for psychology and behavioral economics but also for fields like ethics, policy-making, and artificial intelligence. While much research has examined the choices people make in these scenarios, less is known about the emotional structures that underlie these decisions. Do economic and moral decisions elicit distinct affective patterns? And if so, can these emotional responses predict the nature of the decision-making context?

Research on economic decision-making has highlighted the profound influence of emotions on bargaining behavior, particularly in the ultimatum game. In this paradigm, proposers suggest how to split a sum of money, and responders decide whether to accept or reject offers. Rejections of unfair offers, even at a personal cost, emphasize the role of emotions over pure economic rationality. Studies using biofeedback measures, such as galvanic skin response, have demonstrated heightened physiological arousal in decisions to reject unfair offers, indicating that visceral emotional reactions drive these outcomes (He & Tang, 2017). Recent research further supports this idea, showing that affective responses such as anger and frustration significantly influence bargaining behavior, with increased neural activation in the anterior insula linked to higher rejection rates of unfair offers (Song et al., 2024). Additionally, among alcohol-dependent individuals, greater sensitivity to unfairness correlates with amplified emotional responses, suggesting a link between emotional reactivity and maladaptive decision-making (Brevers et al., 2015). Beyond momentary emotional states, both state and trait emotions modulate ultimatum game behavior. For instance, induced happiness increases acceptance of unfair offers, whereas negative trait affect amplifies rejection tendencies, with these effects reflected in neural markers such as feedback-related negativity and P3b amplitudes, which capture emotional and motivational processing (Riepl et al., 2016). Additionally, proposer facial expressions following a responder’s decision further influence perceived success in enforcing social norms, emphasizing the interplay between socio-emotional stimuli and decision outcomes (Mussel et al., 2022). Previous neuroimaging studies have shown that fairness-related emotional reactions are deeply embedded within core neural circuits, governing economic decision-making across different social contexts (Corradi-Dell’Acqua et al., 2013; Xu et al., 2025; Gilam et al., 2015; Chen et al., 2019). While previous research has examined how individuals’ emotional states influence economic decision-making, less attention has been given to how economic bargaining situations themselves elicit specific emotional responses.

In contrast to economic games, moral dilemmas, such as the trolley problem, require individuals to make decisions about sacrificing one life to save multiple others. Research suggests that emotions play a dual role in moral judgment, influencing both automatic, intuitive processes and deliberate, reflective reasoning. Greene’s dual-process model (Greene, 2007) posits that utilitarian judgments require controlled cognitive processes, whereas deontological responses are driven by automatic emotional aversion to harm (Gothard & Davies, 2024). Experimental studies using virtual trolley dilemmas have demonstrated that autonomic arousal increases in action-based moral dilemmas, leading to a decreased likelihood of utilitarian decisions, suggesting that heightened emotional engagement complicates moral reasoning (Navarrete et al., 2012). More recent evidence indicates that distinct neural responses occur depending on whether the moral dilemma involves direct physical harm versus indirect harm, with increased amygdala activation linked to stronger deontological judgments (Skalski-Bednarz et al., 2025). Interestingly, emotional reactions do not always predict moral judgments. Studies using self-report measures, such as PANAS-X, have found comparable emotional intensities across different versions of the trolley dilemma (e.g., switch vs. footbridge), challenging the assumption that emotions alone drive divergent moral choices (Horne & Powell, 2013). Supporting this view, neuroimaging evidence indicates that moral decision-making recruits a network that balances utilitarian reasoning (e.g., vmPFC; Hu & Jiang, 2014) with emotional aversion to harm (e.g., anterior insula, amygdala; Sarlo et al., 2014). Moreover, early neural markers like the P260 component reflect the temporal conflict between emotional and cognitive processes during moral evaluation (Tang & Lin, 2015). Taken together, these findings suggest a complex interplay between affect and cognition, where emotions play a role but are not the sole drivers of moral decisions (Horne & Powell, 2016).

Despite extensive research on economic and moral decision-making, how these situations shape individuals’ emotional responses, rather than being influenced by their preexisting emotional states, remains unclear. Emotions are crucial in shaping fairness perceptions, moral responsibility, and conflict resolution, yet their underlying affective structure has not been systematically explored. This study seeks to fill this gap by mapping the emotional dimensions of economic and moral decision-making scenarios using multidimensional scaling (MDS) and machine learning classification. By achieving this, we aim to determine whether these decisions evoke distinct and consistent emotional representations and whether these affective patterns can reliably predict scenario types. MDS is particularly useful for identifying latent dimensions that structure affective experiences inferred from data patterns (Shinkareva et al., 2013). However, while MDS reveals underlying affective structures, it does not inherently specify where individual emotions are positioned along these dimensions. To address this, we applied vector fitting, a technique that aligns affective scales with the extracted dimensions to better interpret their spatial representation (Borg & Groenen, 2005). Additionally, we employed machine-learning-based classification analyses to predict scenario types based on affective responses. Classification methods are particularly effective in identifying complex patterns within high-dimensional data, allowing for the prediction of new observations and uncovering latent affective structures that might not be detectable through traditional univariate methods (Haynes & Rees, 2006; Pereira et al., 2009). The primary objective of this study was to investigate how economic and moral decisions are emotionally represented and whether these representations can be systematically predicted using multivariate approaches.

## 2. Methods

### 2.1. Participants

To determine the required sample size, we conducted a power analysis using the GPower software (ver 3.1) (Faul et al., 2007). Based on the effect sizes obtained from our previous studies (Kim & Kim, 2022, 2024; Park et al., 2023; Jo et al., 2023; Park & Kim, 2024a, 2024b; Yang et al., 2024), we aimed to achieve a statistical power of 0.8. Power analysis based on these parameters with the desired power level indicated a sample size of 16. Considering the possibility of missing data, we recruited a total of 23 participants from the university community (6 male, 17 female), and participants provided signed written consent forms in accordance with the Institutional Review Board at the university (approval No. 2024-08-006-002). All participants reported normal vision.

### 2.2. Stimuli

Participants were presented with a total of 16 scenarios in a within-subject design, structured as a 2 (game type: economic—ultimatum game vs. moral—trolley dilemma) × 2 (conflict: conflict vs. no conflict) × 4 (intensity) factorial design. The exact scripts presented to the participants are detailed in Table 1.

The conflict condition in the ultimatum game followed the standard paradigm: another participant acted as the proposer and was given KRW 10,000 to divide between themselves and the participant, who played the role of the responder. The participant was asked whether they would accept or reject the proposer’s offer. If they accepted, both parties would receive the proposed amounts. If they rejected, neither party received any money. The intensity of the conflict was manipulated by varying the fairness of the offer—higher-intensity conditions featured highly unfair offers (e.g., proposer: KRW 8000, participant: KRW 2000), whereas lower-intensity conditions featured more equitable divisions (e.g., proposer: KRW 5000, participant: KRW 5000). In contrast, the no-conflict condition in the ultimatum game involved a scenario where the proposer simply offered money to the participant, without any inherent unfairness. The participant always benefited from accepting the offer, as there was no possible loss. Thus, in this condition, there was no reason for the participant to reject the proposal, eliminating the conflict typically associated with the ultimatum game. A key distinction in our design was the presence or absence of social comparison. In the conflict condition, participants evaluated their allocated amount relative to another’s share, engaging in social comparison even when the division was fair. In contrast, the no-conflict condition presented an amount directly to the participant without reference to another person, eliminating the need for fairness evaluation or distribution concerns.

The conflict condition in the trolley dilemma followed the classical formulation: a runaway trolley was heading toward a track where multiple individuals were tied down. If the participant took no action, these individuals would be killed. However, the participant had the option to pull a lever, redirecting the trolley onto another track, where a single individual was tied down. The intensity of the dilemma was manipulated by altering the number of individuals on the initial track. Higher-intensity conditions featured a smaller number of individuals at risk (e.g., two people), making the decision more difficult, whereas lower-intensity conditions featured a larger number of people at risk (e.g., eight people), making the decision relatively easier. In the no-conflict condition of the trolley dilemma, the basic setup remained the same. There were individuals tied down on the track where the trolley was originally headed. However, in this condition, pulling the lever diverted the trolley to an empty track with no individuals at risk. Thus, making the decision to change the trolley’s direction guaranteed that no one would be harmed, eliminating the moral conflict inherent in the classic dilemma.

### 2.3. Procedure

The experiment was conducted online using Google Forms. On the first page of the survey, participants were provided with a detailed explanation of the study and were asked to indicate their consent to participate. Participants also recorded the time they began the experiment. Those who consented proceeded to the second page, where they provided demographic information, including gender and age. From the third page onward, participants were sequentially presented with one of the sixteen scenarios described in the Section 2.2. For each scenario, participants were instructed to read the description carefully and then rate their emotional responses to ten adjectives (angry, fearful, irritated, happy, calm, peaceful, pleased, anxious, sad, gloomy) using a 7-point Likert scale (1 = not at all, 7 = extremely). After completing the emotional ratings, participants made a decision specific to the scenario type: either accepting or rejecting the proposer’s offer in the ultimatum game or deciding whether to pull the lever to divert the train to the alternative track in the trolley dilemma. This procedure was repeated for all 16 scenarios, ensuring that each scenario and the corresponding adjectives were presented in a randomized order to minimize order effects. On the final page of the survey, participants were provided with a debriefing and instructions to conclude their participation in the study. This structured, iterative procedure allowed for the systematic collection of emotional ratings and decision-making data across all conditions while maintaining a randomized presentation of stimuli and measures.

### 2.4. Statistical Analyses

#### 2.4.1. Multidimensional Scaling (MDS)

Multidimensional scaling (MDS) is a statistical technique used to represent data in a lower-dimensional space while preserving the relationships between objects based on their similarity or dissimilarity. In this study, the “objects” refer to the 16 moral and economic scenarios, and MDS visualizes how these scenarios are positioned relative to each other. The goal is to see whether their arrangement in a simplified space reflects fundamental affective dimensions, such as valence (positive vs. negative feelings) and arousal (intensity of emotional response). Since MDS itself does not test for statistical significance, we applied the Procrustes rotation (Gower, 1975) to align the MDS solution with our study’s design matrix. We then tested the significance of this alignment by examining the correlation between the rotated MDS coordinates and the design values.

A 16 × 16 (stimuli × stimuli) correlation matrix was created for each participant based on their affective ratings, and individual matrices were averaged to produce a group-level correlation matrix. This group matrix was then subjected to three-dimensional MDS, and the resulting solution was Procrustes-rotated to align with the design matrix.

To better understand the relationships between the extracted dimensions and the emotional ratings, vector fitting was performed on the rotated MDS solution (Borg & Groenen, 2005). Each vector represented an affective adjective from the rating scale. The length of a vector indicated the strength of its relationship with the corresponding dimension, while the direction corresponded to the predicted value based on its orthogonal projection. This provided insights into the relative contribution of each dimension. The mean affective ratings for each stimulus were computed by averaging across participants, resulting in an aggregate score for each adjective. Regression analyses were conducted to examine the relationship between the coordinates of each stimulus in the multidimensional space and the mean ratings of the affective adjectives. For each adjective, the coordinates of the stimuli served as the independent variables, while the mean ratings served as the dependent variable. The regression coefficients were used to determine the relative contribution of each dimension, while the coefficient of determination (R^2^) was derived from the length of the fitted vector in the multidimensional space. This process allowed for the visualization of the 10 affective adjectives within the multidimensional space, highlighting their relationship to the extracted dimensions and providing a comprehensive representation of the affective structure underlying the economic and moral decision-making scenarios.

#### 2.4.2. Classifications

Classification analyses were conducted to determine whether the experimental conditions (scenario type, conflict vs. no-conflict, and intensity) could be predicted based on participants’ affective ratings. Two types of classification were performed: within-participant and cross-participant classification. Within-participant classification is performed using data from each individual separately. The classifier is trained on a portion of a participant’s data (training set) and then tested on the remaining portion (test set). Thus, a participant’s classification performance is independent of other participants’ performances. This procedure is conducted for each participant, and the results are typically presented as the average classification accuracy across all participants. Significant classification performance in this analysis would indicate that a participant’s emotional responses contained sufficient information to reliably distinguish between the experimental conditions. In contrast, cross-participant classification utilizes data from all participants together. In this approach, data from one participant serve as the test set, while data from the remaining participants serve as the training set. The classifier is trained on the training set and then tested on the test set to predict the experimental conditions. Even if all participants demonstrate significant within-participant classification performance, this does not necessarily guarantee significant cross-participant classification performance. Each participant is tested once in this framework, and the final result is presented as the average classification accuracy across all participants. Significant classification performance in this analysis would suggest consistency in emotional responses across participants, allowing for the experimental conditions to be accurately predicted from affective ratings regardless of individual differences.

For the within-participant classification, the affective ratings for one scenario served as the test set, while the ratings for the remaining 15 scenarios were used as the training set. A Support Vector Machine (SVM) classifier was trained using the training set and subsequently tested on the test set. Classification accuracy was calculated by averaging the results from sixteen cross-validation folds, each corresponding to one scenario being used as the test set. Significant classification performance would indicate that the participant’s emotional responses contained sufficient information to reliably distinguish between the experimental conditions.

For the cross-participant classification, the affective ratings of one participant were used as the test set, while the ratings of the remaining participants were used as the training set. The SVM classifier, trained on the training data, was evaluated using the test set. The classification accuracy was computed as the average performance across twenty-three cross-validation folds, where each fold corresponded to one participant being held out as the test set. Significant classification performance in this analysis would suggest consistency in emotional responses across participants, allowing for the experimental conditions to be accurately predicted from affective ratings regardless of individual differences.

These classification procedures enabled the investigation of both individual-level and group-level consistency in affective responses, providing insight into whether the experimental conditions were systematically encoded in the participants’ emotional expressions.

## 3. Results

### 3.1. MDS

**Figure 1:**
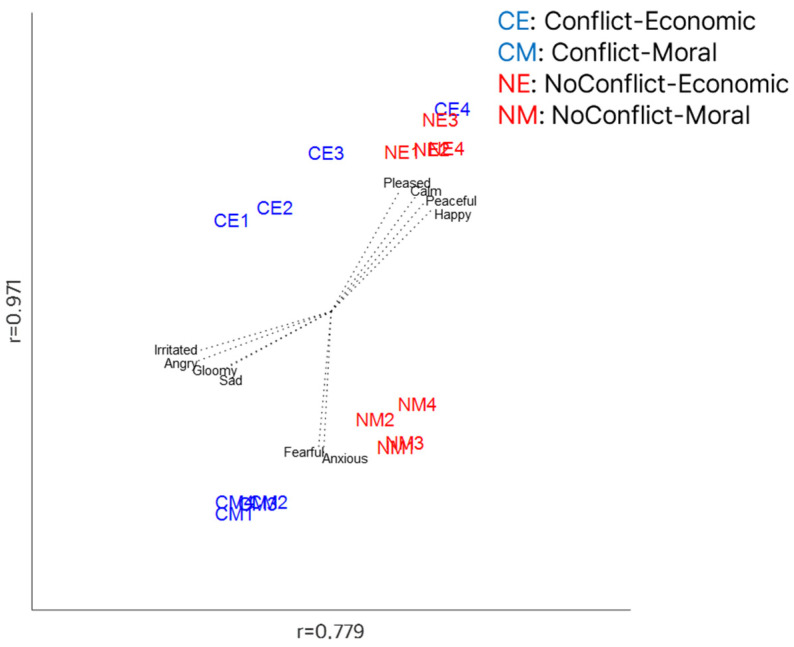
The results of rotated three-dimensional MDS with the vector fitting of the economic and moral decision-making scenarios. Each vector indicates the adjectives used to rate the affective responses of the scenarios. r = Pearson’s correlation coefficient between the coordinates of the rotated MDS solution and the design values.

The economic and moral decision-making scenarios were represented in a three-dimensional space (Figure 1). Pearson’s correlation analyses were conducted to examine the relationship between the design matrix and the coordinates of the rotated MDS solution for each dimension. The first dimension, representing conflict versus no-conflict, was significant (r = 0.779, *p* < 0.05), indicating substantial differences in affective states between the conflict and no-conflict scenarios. The second dimension, reflecting economic versus moral scenarios, was also significant (r = 0.971, *p* < 0.05), suggesting clear distinctions in affective states between economic and moral decision-making contexts. In contrast, the third dimension, associated with intensity, was not significant (r = 0.348, *p* > 0.05). For additional validity and reliability analyses, including the multitrait–multimethod matrix and correlation matrix of the scenarios, please refer to Supplementary Tables S1 and S2.

The conflict–economic scenario 4 (where the proposer and participant split the money equally at KRW 5000 each) was positioned in the same region as the no-conflict–economic scenarios. Unlike the other conflict scenarios, the conflict–economic scenario 4 involves both individuals receiving money, but since the split is perfectly equal (KRW 5000/KRW 5000), there is no perceived unfairness. This similarity in fairness likely explains why it aligned with the no-conflict–economic scenarios. Although the intensity dimension was not statistically significant, the positioning of the conflict–economic scenarios suggests that as unfairness increases, these scenarios diverge further from the no-conflict–economic scenarios. In other words, the fairer the conflict–economic scenario feels, the closer it is perceived to be to a no-conflict situation. This indicates that the no-conflict–economic scenarios may exhibit some effects of intensity. In contrast, for the conflict–moral scenarios, there was little difference in affective representation regardless of whether the dilemma involved 1 person vs. 2 people or 1 person vs. 8 people. This suggests that intensity has no meaningful effect on how participants perceive moral dilemmas. These findings highlight the nuanced role of fairness and intensity in shaping the affective structure of economic and moral decision-making scenarios.

To further interpret the relationship between the affective ratings and the extracted dimensions, vector fitting was applied to the rotated MDS solution. The results revealed that the no-conflict–economic scenarios were associated with more positive affective ratings, particularly pleased, calm, happy, and peaceful, compared to other scenarios. Interestingly, contrary to expectations, the no-conflict–moral scenarios did not elicit strongly positive affective responses. Instead, they were closely associated with scales such as fearful and anxious. Both conflict scenarios (conflict–economic and conflict–moral) were predominantly linked to negative affective states, with irritated, angry, gloomy, and sad being the most prominent. The findings from the MDS analysis provide a clear visualization of how economic and moral decision-making scenarios are represented within the core affect dimension of valence.

### 3.2. Classifications

**Figure 2:**
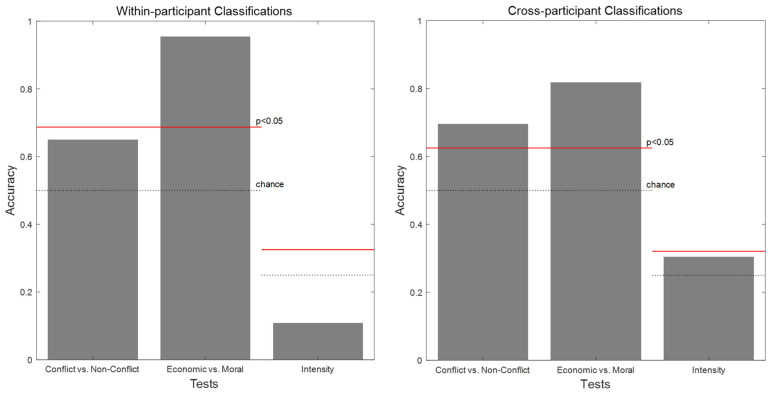
The results of the within- (**left**) and cross-participant classification (**right**). Each bar of the graphs indicates the accuracy of each test. The dotted lines indicate the chance level, and the solid red lines indicate the critical value.

The within-participant and cross-participant classification analyses demonstrated that the experimental conditions of the economic and moral decision-making scenarios could be significantly predicted based on affective ratings (Figure 2). In the within-participant classification results (Figure 2, left), the prediction of economic vs. moral scenarios was significant, whereas the prediction of conflict vs. no-conflict scenarios was not significant. Additionally, the classification of intensity was not significant. In the cross-participant classification results (Figure 2, right), both conflict vs. no-conflict and economic vs. moral scenarios were significantly classified. However, similar to the within-participant analysis, intensity was not classified at a significant level. Given that cross-participant classification is generally more challenging than within-participant classification due to individual differences in affective responses and the added variability introduced by using different participants’ data as the training set, the non-significant result for conflict vs. no-conflict in the within-participant analysis may be attributed to the relatively small size of the training set (15 trials). In summary, these findings indicate that the experimental conditions related to decision type and the presence or absence of conflict can be reliably predicted from affective ratings. Furthermore, the consistency of these results across participants suggests that the affective responses underlying these classifications are robust and shared across individuals.

### 3.3. Acceptance Rates

The participants were asked to either accept or reject economic offers in the ultimatum game and to choose whether or not to pull the lever in moral dilemmas. Acceptance rates indicate the proportion of trials in which the participants chose the “accept” or “action” option. The mean acceptance rates were conflict–economic = 0.7174, no-conflict–economic = 0.7717, conflict–moral = 0.8696, and no-conflict–moral = 0.9891. A two-way ANOVA revealed a significant main effect of decision type (economic vs. moral), *F*(1, 22) = 5.89, *p* = 0.024, indicating that the participants were more likely to accept moral decisions than economic decisions. The main effect of conflict (conflict vs. no-conflict) was not statistically significant, *F*(1, 22) = 4.16, *p* = 0.054. Although not statistically significant, the pattern aligned with our expectations. The participants were more likely to accept proposals or to turn the lever in the no-conflict condition than in the conflict condition. The interaction effect was also not significant, *F*(1, 22) = 0.570, *p* = 0.458. Within the economic scenarios, the acceptance rate was higher in the no-conflict condition than in the conflict condition, but this difference was not statistically significant, *t*(22) = −0.894, *p* = 0.381. Within the moral scenarios, the difference between the conflict and no-conflict conditions was larger than in the economic scenarios, but again, this difference did not reach statistical significance, *t*(22) = −1.972, *p* = 0.061.

## 4. Discussion

This study investigated the affective structure underlying economic and moral decision-making scenarios using an ultimatum game and trolley dilemma. A total of 16 scenarios were presented to participants, who rated their emotional responses on affective scales. The MDS results revealed that the first dimension significantly distinguished conflict from no-conflict scenarios, while the second dimension separated economic from moral scenarios. The classification analyses successfully predicted experimental conditions based on affective ratings.

This study provides novel insights into the affective organization of economic and moral decision-making scenarios, revealing a structured representation along conflict and game type dimensions. Unlike prior research that primarily focused on binary choices in these paradigms, this study uniquely quantified emotional responses and leveraged multidimensional scaling and machine learning to uncover latent patterns in affective space. The findings highlight that perceived fairness in economic exchanges can reduce conflict perception, blurring the boundary between conflict and non-conflict conditions in economic scenarios, while moral dilemmas elicit a consistent affective structure regardless of intensity variations. Moreover, the successful classification of decision type (economic vs. moral) and conflict presence across the participants suggests that affective responses to decision-making are systematic and generalizable rather than idiosyncratic. These results extend previous work by demonstrating that affective representations, rather than explicit choices alone, serve as reliable markers of decision-making contexts.

### 4.1. Interpretation of Key Findings

One interesting result was that fair conflict–economic scenarios aligned more closely with no-conflict–economic scenarios, while intensity did not differentiate moral scenarios. The differential affective responses to economic and moral dilemmas can be understood through the lens of fairness sensitivity and intensity effects. While economic dilemmas elicit stronger affective responses as unfairness increases, moral dilemmas appear to evoke a relatively stable emotional reaction regardless of the number of lives at stake. This discrepancy can be attributed to fundamental differences in how individuals process economic versus moral decision-making scenarios. Economic decision-making is deeply influenced by considerations of fairness, which is supported by the concept of inequity aversion (Fehr & Schmidt, 1999). When monetary distributions are perceived as unequal, individuals experience heightened emotional conflict, particularly when the degree of unfairness is pronounced. Studies on the ultimatum game further support this notion, demonstrating that individuals are willing to reject unfair economic offers despite incurring personal financial losses, indicating that fairness violations strongly shape affective responses in economic contexts (Sanfey et al., 2003). The optimization–fairness trade-off (Banerjee & Veltri, 2024) highlights that individuals strive to balance economic efficiency with fairness, leading to stronger affective responses in scenarios where monetary allocations are highly unequal. Kahneman and Tversky’s (1979) Prospect Theory also aligns with this, showing that people are highly sensitive to numerical differences in economic transactions, particularly in losses and gains.

In contrast, moral dilemmas involving human lives are processed through a fundamentally different cognitive framework. Unlike economic decisions, where numerical differences in allocations significantly impact emotional responses, moral decisions tend to be guided by deontological reasoning, which categorically values human life (Greene et al., 2001). Studies on behavioral economics (Thaler, 1980) highlight that economic decisions involve systematic cost–benefit calculations, meaning that numerical differences affect decision-making intensity. However, research in moral psychology has demonstrated that people often exhibit scope insensitivity, meaning that their emotional response does not proportionally scale with the number of lives affected (Fetherstonhaugh et al., 1997). In scenarios where individuals must decide between sacrificing one versus two lives, or one versus eight lives, the affective conflict remains relatively stable, suggesting that human life is evaluated in a qualitative rather than quantitative manner. Greene et al. (2004) further distinguished between cognitive utilitarian moral judgments, which require deliberate reasoning, and intuitive deontological judgments, which are fast and emotional. This intuitive resistance to quantifying human life results in a ceiling effect, where affective responses remain stable despite increasing numerical stakes. While fairness violations in economic dilemmas elicit graded affective responses due to intensity effects, moral dilemmas remain relatively unaffected by numerical scaling, as human life is perceived as categorically distinct from economic trade-offs. These findings underscore the nuanced role of fairness and intensity in shaping affective decision-making. While economic dilemmas elicit stronger negative responses, including anger, as fairness violations increase, moral dilemmas elicit a stable level of anxiety and fear in affective intensity due to deep-seated moral intuitions. This divergence highlights the importance of considering context-dependent cognitive frameworks when analyzing decision-making behavior across different domains.

The participants showed a higher acceptance rate in moral scenarios than in economic ones, although the effect of conflict was not statistically significant. These behavioral patterns complement the affective findings, suggesting that the participants were more inclined to act in moral contexts, possibly due to strong internalized moral norms. The lack of significant differences across conflict conditions may indicate that affective responses did not directly determine behavioral outcomes.

### 4.2. The Role of Fear and Anxiety in Moral Decision-Making

Another interesting result was that the no-conflict–moral scenarios were closely associated with scales such as fearful and anxious. While it may be expected that moral scenarios with clear and positive outcomes would evoke minimal distress, empirical findings indicate that such situations are instead associated with heightened levels of fear and anxiety. Kurth (2015) argues that moral anxiety is an intrinsic part of ethical deliberation, emerging when individuals confront decisions that have moral weight. Even in the no-conflict–moral scenarios in the current study, where no harm results from the decision, individuals might experience anxiety due to uncertainty regarding the correctness of their choice. This anxiety could stem from an implicit concern about whether the action aligns with moral norms and societal expectations. As a result, rather than experiencing relief, individuals may feel a heightened sense of responsibility, potentially leading to increased stress and cognitive burden. Also, Tao et al. (2023) suggested that fear plays a significant role in moral decision-making by increasing cognitive deliberation and caution. In a moral judgment task, participants experiencing fear were more likely to engage in systematic reasoning and showed stronger physiological reactions when making decisions. This finding implies that even in cases where harm is avoidable, individuals remain in a heightened state of emotional vigilance, fearing potential unknown consequences or the moral implications of inaction. Merlhiot et al. (2018) highlighted the role of uncertainty in increasing emotional arousal during moral decision-making. While no-conflict–moral scenarios seem straightforward, they still contain an element of uncertainty such as “What if I do not act?” or “Am I absolutely sure that pulling the lever is the best choice?” This latent uncertainty may lead to heightened emotional responses, as the individual struggles with the cognitive dissonance between an apparently clear decision and underlying doubts about the action’s moral justification. Wu et al. (2013) demonstrated that individuals with higher anxiety levels show stronger affective reactions in social decision-making contexts. In this type of scenario, although no harm may occur, the decision-maker may feel full responsibility for the outcome. This sense of personal responsibility, combined with the perception of moral accountability, may explain why participants exhibit fear and anxiety rather than relief or positive emotions. Overall, the findings from prior research suggest that moral decision-making is deeply intertwined with affective responses, even in scenarios where harm is preventable. Contrary to expectations, the no-conflict–moral scenarios in this study did not evoke positive emotions. Instead, these scenarios may elicit significant levels of fear and anxiety, possibly due to moral anxiety, increased cognitive deliberation, uncertainty, and perceived social responsibility. These findings suggest that emotions play a crucial role in ethical reasoning and highlight potential directions for further research on moral cognition.

### 4.3. Limitations of the Study

One key limitation of this study is that all the decisions were made in hypothetical scenarios rather than real-life situations involving actual monetary gains or life-and-death consequences. This lack of tangible stakes may have influenced the participants’ emotional responses and decision-making processes, potentially reducing ecological validity. Additionally, while the study effectively captured affective representations of decision-making contexts, it did not assess reaction times or physiological responses, which could provide further insights into cognitive and emotional processing. Furthermore, the reliance on self-reported emotions introduces potential biases, as the participants may have been influenced by social desirability or demand characteristics.

Finally, although the present study did not perform a factor analysis on the affective scale responses, this was a deliberate methodological decision, as our primary focus was on the relational structure of scenarios in affective space using MDS. Nonetheless, we acknowledge that future studies could benefit from examining the underlying factor structures across different decision-making contexts, which may yield complementary insights into the dimensionality of affective responses.

### 4.4. Recommendations for Future Research

Future research should explore real-world decision-making contexts by incorporating actual monetary incentives or immersive virtual reality simulations to enhance ecological validity. Additionally, integrating physiological measures such as heart rate variability, skin conductance, or neuroimaging could provide deeper insights into the neural and autonomic processes underlying affective responses. Given the non-significant effect of intensity in moral dilemmas, future studies should refine intensity manipulations by considering emotional proximity (e.g., sacrificing a loved one vs. a stranger) or moral responsibility (e.g., personal involvement vs. passive observation).

## Figures and Tables

**Table 1:** The 16 scenarios describing 2 (game type: economic—ultimatum game vs. moral—trolley dilemma) × 2 (conflict: conflict vs. no conflict) × 4 (intensity).

	Intensity	Ultimatum Game	Trolley Dilemma
Conflict	1	You are the responder, and another participant acts as the proposer. The proposer is given KRW 10,000 and offers to divide it such that you receive KRW 2000, while they keep KRW 8000. You can either accept or reject the offer. If you accept, both receive the proposed amounts. If you reject, neither receives anything.	You are operating an unstoppable train heading toward a fork. On the left track, 2 workers are tied down, and on the right track, 1 worker is tied down. If you do nothing, the train will continue on the left track, killing 2 workers. The only way to save them is to pull the lever to divert the train to the right track, which will result in 1 worker’s death.
	2	You are the responder, and another participant acts as the proposer. The proposer is given KRW 10,000 and offers to divide it such that you receive KRW 3000, while they keep KRW 7000. You can either accept or reject the offer. If you accept, both receive the proposed amounts. If you reject, neither receives anything.	You are operating an unstoppable train heading toward a fork. On the left track, 4 workers are tied down, and on the right track, 1 worker is tied down. If you do nothing, the train will continue on the left track, killing 4 workers. The only way to save them is to pull the lever to divert the train to the right track, which will result in 1 worker’s death.
	3	You are the responder, and another participant acts as the proposer. The proposer is given KRW 10,000 and offers to divide it such that you receive KRW 4000, while they keep KRW 6000. You can either accept or reject the offer. If you accept, both receive the proposed amounts. If you reject, neither receives anything.	You are operating an unstoppable train heading toward a fork. On the left track, 6 workers are tied down, and on the right track, 1 worker is tied down. If you do nothing, the train will continue on the left track, killing 6 workers. The only way to save them is to pull the lever to divert the train to the right track, which will result in 1 worker’s death.
	4	You are the responder, and another participant acts as the proposer. The proposer is given KRW 10,000 and offers to divide it equally: KRW 5000 for you and KRW 5000 for themselves. You can either accept or reject the offer. If you accept, both receive the proposed amounts. If you reject, neither receives anything.	You are operating an unstoppable train heading toward a fork. On the left track, 8 workers are tied down, and on the right track, 1 worker is tied down. If you do nothing, the train will continue on the left track, killing 8 workers. The only way to save them is to pull the lever to divert the train to the right track, which will result in 1 worker’s death.
No-Conflict	1	You are the responder, and another participant acts as the proposer. The proposer offers KRW 2000 to you with no further division involved. You can either accept or reject the offer. Accepting gives you the proposed amount, while rejecting results in you receiving nothing.	You are operating an unstoppable train heading toward a fork. On the left track, 2 workers are tied down, and the right track is empty. If you do nothing, the train will continue on the left track, killing 2 workers. The only way to save them is to pull the lever to divert the train to the empty right track.
	2	You are the responder, and another participant acts as the proposer. The proposer offers KRW 3000 to you with no further division involved. You can either accept or reject the offer. Accepting gives you the proposed amount, while rejecting results in you receiving nothing.	You are operating an unstoppable train heading toward a fork. On the left track, 4 workers are tied down, and the right track is empty. If you do nothing, the train will continue on the left track, killing 4 workers. The only way to save them is to pull the lever to divert the train to the empty right track.
	3	You are the responder, and another participant acts as the proposer. The proposer offers KRW 4000 to you with no further division involved. You can either accept or reject the offer. Accepting gives you the proposed amount, while rejecting results in you receiving nothing.	You are operating an unstoppable train heading toward a fork. On the left track, 6 workers are tied down, and the right track is empty. If you do nothing, the train will continue on the left track, killing 6 workers. The only way to save them is to pull the lever to divert the train to the empty right track.
	4	You are the responder, and another participant acts as the proposer. The proposer offers KRW 5000 to you with no further division involved. You can either accept or reject the offer. Accepting gives you the proposed amount, while rejecting results in you receiving nothing.	You are operating an unstoppable train heading toward a fork. On the left track, 8 workers are tied down, and the right track is empty. If you do nothing, the train will continue on the left track, killing 8 workers. The only way to save them is to pull the lever to divert the train to the empty right track.

## Data Availability

The raw data supporting the conclusions of this article will be made available by the corresponding author upon reasonable request.

## Supplementary Materials

The following supporting information can be downloaded at: https://www.mdpi.com/article/10.3390/bs15040558/s1, Table S1. The multitrait-multimethod matrix. Off-diagonal values represent Pearson correlation coefficients (r), while diagonal values indicate Cronbach’s alpha. Table S2. Correlation matrix of economic and moral scenarios.
