# Supplementary material for: Beyond Choice: Affective Representations of Economic and Moral Decisions
Source: Behav Sci (Basel). 2025 Apr 21;15(4):558. doi: 10.3390/bs15040558 (PMC12025302; doi:10.3390/bs15040558)
Supplement: Supplementary file 1 [file behavsci-15-00558-s001.zip › behavsci-3503933-supplementary.pdf]

Table S1. The multitrait-multimethod matrix. Off-diagonal values represent Pearson correlation coefficients (r), while diagonal values indicate Cronbach's alpha.

	Sad	Angry	Calm	Anxious	Annoyed	Melancholic	Happy	Relaxed	Fearful	Pleased
Sad	0.89									
Angry	0.67	0.83								
Calm	-0.65	-0.67	0.89							
Anxious	0.7	0.58	-0.73	0.87						
Annoyed	0.6	0.83	-0.64	0.58	0.79					
Melancholic	0.77	0.59	-0.57	0.65	0.61	0.89				
Happy	-0.52	-0.57	0.63	-0.53	-0.58	-0.5	0.87			
Relaxed	-0.63	-0.7	0.88	-0.72	-0.68	-0.58	0.72	0.88		
Fearful	0.71	0.58	-0.74	0.89	0.55	0.64	-0.56	-0.72	0.86	
Pleased	-0.48	-0.51	0.65	-0.51	-0.54	-0.47	0.86	0.71	-0.54	0.85

Table S2. Correlation matrix of economic and moral scenarios.

	CE1	CE2	CE3	CE4	NE1	NE2	NE3	NE4	CM1	CM2	CM3	CM4	NM1	NM2	NM3	NM4
CE1	1															
CE2	0.65	1														
CE3	0.52	0.57	1													
CE4	0.12	0.16	0.56	1												
NE1	0.39	0.48	0.58	0.49	1											
NE2	0.29	0.4	0.61	0.68	0.74	1										
NE3	0.24	0.35	0.67	0.78	0.73	0.85	1									
NE4	0.16	0.24	0.64	0.82	0.61	0.74	0.78	1								
CM1	0.05	-0.03	-0.29	-0.7	-0.36	-0.46	-0.57	-0.52	1							
CM2	0.01	0.01	-0.32	-0.7	-0.29	-0.44	-0.52	-0.54	0.83	1						
CM3	0.04	0.03	-0.26	-0.66	-0.31	-0.42	-0.5	-0.49	0.9	0.85	1					
CM4	0.06	0.03	-0.26	-0.68	-0.34	-0.44	-0.53	-0.52	0.9	0.82	0.92	1				
NM1	-0.02	-0.05	-0.05	-0.13	-0.09	-0.01	-0.13	0.01	0.38	0.34	0.41	0.38	1			
NM2	0.09	0.06	0.01	-0.04	-0.04	0.03	-0.06	0.04	0.33	0.22	0.33	0.28	0.77	1		
NM3	0.01	0.05	-0.05	-0.23	0	-0.02	-0.08	-0.07	0.48	0.48	0.54	0.48	0.64	0.62	1	
NM4	0.04	0.05	0.05	0.05	0.04	0.13	0.02	0.11	0.23	0.15	0.25	0.19	0.69	0.82	0.66	1
